# Current Advances on Virus Discovery and Diagnostic Role of Viral Metagenomics in Aquatic Organisms

**DOI:** 10.3389/fmicb.2017.00406

**Published:** 2017-03-22

**Authors:** Hetron M. Munang'andu, Kizito K. Mugimba, Denis K. Byarugaba, Stephen Mutoloki, Øystein Evensen

**Affiliations:** ^1^Section of Aquatic Medicine and Nutrition, Department of Basic Sciences and Aquatic Medicine, Faculty of Veterinary Medicine and Biosciences, Norwegian University of Life SciencesOslo, Norway; ^2^Department of Biotechnical and Diagnostic Sciences, College of Veterinary Medicine, Animal Resources and Biosecurity, Makerere UniversityKampala, Uganda

**Keywords:** aquatic organisms, diagnosis, discovery, etiology, novel, viral metagenomics, viruses

## Abstract

The global expansion of the aquaculture industry has brought with it a corresponding increase of novel viruses infecting different aquatic organisms. These emerging viral pathogens have proved to be a challenge to the use of traditional cell-cultures and immunoassays for identification of new viruses especially in situations where the novel viruses are unculturable and no antibodies exist for their identification. Viral metagenomics has the potential to identify novel viruses without prior knowledge of their genomic sequence data and may provide a solution for the study of unculturable viruses. This review provides a synopsis on the contribution of viral metagenomics to the discovery of viruses infecting different aquatic organisms as well as its potential role in viral diagnostics. High throughput Next Generation sequencing (NGS) and library construction used in metagenomic projects have simplified the task of generating complete viral genomes unlike the challenge faced in traditional methods that use multiple primers targeted at different segments and VPs to generate the entire genome of a novel virus. In terms of diagnostics, studies carried out this far show that viral metagenomics has the potential to serve as a multifaceted tool able to study and identify etiological agents of single infections, co-infections, tissue tropism, profiling viral infections of different aquatic organisms, epidemiological monitoring of disease prevalence, evolutionary phylogenetic analyses, and the study of genomic diversity in quasispecies viruses. With sequencing technologies and bioinformatics analytical tools becoming cheaper and easier, we anticipate that metagenomics will soon become a routine tool for the discovery, study, and identification of novel pathogens including viruses to enable timely disease control for emerging diseases in aquaculture.

## Introduction

Traditionally, the diagnosis of viral diseases has been dependent on cell culture in which viruses exhibit cytopathic effects (CPE) and immunoassays based on antibodies having the binding specificity for the virus to be diagnosed (Leland and Ginocchio, 2007) in addition to amplification of specific gene segments of the viruses by polymerase chain reaction (PCR). Virus propagation using cell-culture has significantly contributed to the development of protective vaccines against fish diseases such as infectious pancreatic necrosis virus (IPNV), viral hemorrhagic septicemia virus (VHSV), infectious hematopoietic necrosis virus (IHNV), salmonid alphavirus (SAV), and infectious salmon anemic virus (ISAV) in aquaculture (Sommerset et al., 2005; Gomez-Casado et al., 2011; Munang'andu et al., 2014a,b; Munang'andu and Evensen, 2015). However, the bulk of viruses found in aquatic environments are unculturable (Wang et al., 2002) and there are no antibodies available for their identification nor specific primers for PCR detection, which precludes their study. The detection of non-culturable viruses (Handelsman, 2004; Schloss and Handelsman, 2005), by molecular biology based tools such as the PCR test demand that the genomic sequence of the target virus be known prior to diagnosis (Yamaguchi et al., 2000; Bibby, 2013), which precludes the identification of novel viruses whose sequences are unknown (Gao and Moore, 1996). Given that aquaculture is continuously faced with the challenge of identifying novel pathogenic viruses infecting different fish, crustaceans, and shellfish species, there is an urgent need to develop diagnostic tools able to identify those viruses in order to expedite the process of developing timely disease control strategies.

In the last decade, high throughput next generation sequencing (NGS), has emerged not only as a powerful tool able to enhance our understanding of the host response to infection (Xu et al., 2015, 2016a,b), but as a tool able to unravel a large number of viral genomes using metagenomics analysis (Handelsman, 2004; Riesenfeld et al., 2004). Viral metagenomics analysis is a culture independent approach that does not require prior knowledge of the genomic sequence of the virus to be identified (Handelsman et al., 1998; Streit and Schmitz, 2004; Gross et al., 2010; Mokili et al., 2012; Martínez-Porchas and Vargas-Albores, 2015). It provides a unique opportunity able to identify several viruses in one sample at the same time. The scope of its application in aquaculture is bound to expand from the analysis of environmental microbial composition to the search for novel viruses, routine diagnosis, disease surveillance, and public health. Given the increase of emerging pathogenic viruses in aquaculture, it is anticipated that viral metagenomics is bound to expedite the process of identifying novel viruses that infect different aquatic organisms before they cause disease outbreaks reaching epidemic proportions. The number of viral metagenomics studies has exponentially increased from <10 publications in 2002, when this technology was first discovered (Handelsman, 2004; Riesenfeld et al., 2004), to >300 publications by 2010 (Mokili et al., 2012). Although its application in environmental studies has been widely reviewed by different scientists (Fuhrman, 1999; Breitbart et al., 2002; Edwards and Rohwer, 2005), there are limited reviews on its application in the discovery of novel viruses in aquaculture.

Hence, in this review we first provide a historical background of viral diseases discovered using the traditional cell culture, immunoassays, and basic PCR techniques in aquaculture. As a second step, we provide an overview of viral diseases discovered using viral metagenomics and NGS in order to compare the traditional approaches and viral metagenomics analysis in the discovery of novel viruses. In addition, we also highlight the diagnostic role and other potential uses of viral metagenomics apart from its use in the discovery of novel viruses. Based on observations herein, we advocate that scientists faced with the challenge of identifying new viruses infecting aquatic organisms should explore its application in order to expedite the process of developing timely disease control strategies for emerging viral diseases for aquatic organisms.

## Historical perspectives of viral diseases in aquaculture

The major viral diseases of aquatic organisms can be divided into viruses of finfish, crustaceans, and marine mammals as shown below.

### Viral diseases of finfish

The major challenge in the control of viral diseases in aquaculture is the long duration it takes from the first time the disease is discovered through clinical reports to identification of the etiological agent. This delay precludes our ability to develop timely disease control strategies. For example, IPNV was first reported as a clinical disease causing acute catarrhal enteritis in salmonids in 1940 (McGonigle, 1941) and yet the virus was first identified and characterized 20 years later in 1960 (Wolf et al., 1960). VHSV was first identified in 1962 (Jensen, 1965) followed by IHNV in 1969 (Wingfield et al., 1969) although reports of mortalities caused by both viruses date far back in the early 1950s. In the case of nodavirus, infections causing major problems in hatcheries as well as clinical signs linked to lethargy, anorexia, pale coloration and corkscrew swimming in barramundi (*Lates calcarifer*) and other fish species in Asia were first reported in the 1970s and yet the virus was first characterized in 1990 (Glazebrook et al., 1990; Munday et al., 2002). SAV was first reported as a disease causing skeletal muscle and cardiac myopathy in salmonids in 1976 and yet the virus was first identified and characterized almost two decades later in 1995 (Boucher et al., 1994; Castric et al., 1997). Outbreaks of cardiomyopathy syndrome (CMS) were first reported in 1985 (Amin and Trasti, 1988) while the virus responsible for the disease was recently characterized as piscine myocarditis syndrome virus (PMCV) in 2011 (Haugland et al., 2011). Similarly, heart and skeletal muscle inflammation (HSMI) outbreaks were first reported in 1999 (Kongtorp et al., 2004a,b) and yet the etiological agent was recently identified after a decade as piscine reovirus (PRV) now referred to as piscint orthoreovirus (Løvoll et al., 2010; Palacios et al., 2010). Table 1 shows a summary of the major fish viral diseases showing the year when clinical cases were first reported and the year when the etiological agent was identified. The general trend is that etiological agents were only identified after they caused disease outbreaks reaching epidemics proportions, which is in line with observations made by Alavandi and Poornima (2012) who pointed out that our response to viral infection has mainly been reactive in the sense that a new pathogen is usually not identified until it has reached epidemic proportions. Hence, there is need to develop proactive diagnostic tools able to identify novel viruses before they cause disease outbreaks leading to high economic losses in aquaculture.

**Table 1:** **Viruses infecting finfish discovered using traditional cell culture methods**.

**Virus**	**Family**	**Nucleic acid**	**Clinical signs first reported**		**Etiological virus identified and characterized**	
			**Year**	**References**	Year^*^	**References**
**A: VIRUSES INFECTING FINFISH**						
Infectious pancreatic necrosis virus	Birnaviridae	dsRNA	1940	McGonigle, 1941	1960	Wolf et al., 1960
Viral hemorrhagic septicemia virus	Rhabdoviridae	(−)ssRNA	1950s	Jensen, 1965	1962	Jensen, 1965
Infectious hematopoietic necrosis virus	Rhabdoviridae	(−)ssRNA	1950s	Rucker et al., 1953	1969	Wingfield et al., 1969
Nervous necrosis virus	Nodaviridae	(+)ssRNA	1970s	MacKinnon, 1987	1990	Glazebrook et al., 1990; Mori et al., 1992
Salmonid alphavirus	Togaviridae	(+)ssRNA	1976	Munro et al., 1984	1995	Boucher et al., 1994; Castric et al., 1997
Infectious salmon anemia virus	Orthomyxoviridae	(−)ssRNA	1984	Thorud and Djupvik, 1988	1995	Dannevig et al., 1995; Mjaaland et al., 1997
Hirame rhabdovirus	Rhabdoviridae	(−)ssRNA	1984	Kimura et al., 1986	1984	Kimura et al., 1986
Piscine myocarditis syndrome virus	Totiviridae	dsRNA	1985	Amin and Trasti, 1988	2010	Løvoll et al., 2010; Haugland et al., 2011
Epizootic hematopoietic necrosis virus	Iridoviridae	dsDNA	1985	Langdon et al., 1986	1985	Langdon et al., 1986
Spring viremia of carp virus	Rhabdoviridae	(−)ssRNA	2002	Dikkeboom et al., 2004	2002	Dikkeboom et al., 2004
Tilapia lake virus	Orthomyxoviridae	(−)ssRNA	2009	Eyngor et al., 2014	2014	Eyngor et al., 2014

* *Year virus discovered and characterized*.

### Viral diseases of crustaceans

Apart from fish farming, shrimp farming is one of the rapidly expanding aquaculture industries whose expansion has brought with it a corresponding increase in the number of novel pathogenic viruses being discovered. Similar to observations in fish, Table 2 shows that the year when the first clinical case was identified in shrimp and the year when the etiological agent was identified, depicting long intervals allowing the diseases to reach epidemic levels before the causative agents were identified. For example, mortality due to Taura syndrome (TS) reached high epidemic proportions spreading from the Taura river in Ecuador, where the disease was first reported (Jimenez, 1992), expanding to Peru, Colombia, Brazil, Honduras, and Hawaii. It spread to Asia where it infected different shrimp species before the virus was identified (Walker and Winton, 2010). The cause was initially attributed to fungicide toxicity (Lightner et al., 1994) and the subsequent identification of Taura syndrome virus (TSV) as the etiological agent of the disease led to development of molecular diagnostic tools that paved way to breeding and cultivation of specific pathogen free (SPF) stocks to ensure that all stocks used in shrimp farming were free of the disease (OIE, 2016). Similarly, white spot syndrome virus (WSSV), shrimp infectious myonecrosis virus (IMNV), *Penaeus vannamei* nodavirus (PvNV) and several other shrimp viral diseases reached high epidemic levels before their etiological agents were identified and characterized (Lotz, 1997; Arcier et al., 1999; Tang and Lightner, 1999; Yang et al., 2001; van Hulten et al., 2001). Identification of the causative agents for these diseases paved way to developing diagnostic tools that are currently used for the screening of breeding stocks to ensure that only SPF stocks are used for shrimp production (Lotz, 1997; Arcier et al., 1999; Tang and Lightner, 1999; van Hulten et al., 2001; Yang et al., 2001; OIE, 2016). Hence, it is important that proactive diagnostic tools able to timely identify novel pathogenic viruses are devised in order to expedite the process of developing effective disease control strategies against emerging viral disease in aquaculture.

**Table 2:** **Shrimp viruses discovered using traditional cell culture methods**.

**Virus**	**Virus family**	**Nucleic acid**	**Clinical signs first reported**		**Etiological virus identified and characterized**	
			Year^*^	**References**	Year^*^	**References**
Infectious hypodermal and hematopoietic necrosis virus	Parvoviridae	ssDNA	1981	Brock et al., 1983; Lightner et al., 2004	1984	Lightner and Redman, 1985
Taura syndrome virus	Dicistroviridae	(+)ssRNA	1991	Jimenez, 1992	1994	Hasson et al., 1995
Yellow head disease virus	Roniviridae	(+)ssRNA	1991	Limsuwan, 1991	1999	Tang and Lightner, 1999
White spot syndrome virus	Nimaviridae	dsDNA	1992	Lotz, 1997	2001	van Hulten et al., 2001; Yang et al., 2001
Macrobrachium rosenbergil nodavirus	Nodaviridae	(+)ssRNA	1997	Arcier et al., 1999	1999	Arcier et al., 1999
Shrimp infectious myonecrosis virus	Totiviridae	dsRNA	2002	Lightner et al., 2004; Nunes et al., 2004	2006	Poulos et al., 2006
Penaeus vannamei nodavirus	Nodaviridae	(+)ssRNA	2004	Tang et al., 2007	2004	Tang et al., 2007

* *Year virus discovered and characterized*.

### Marine mammal viral diseases

Although calicivirus infections date as far back as 1932 when pigs that were fed raw garbage in Los Angeles in California resulted in an outbreak that caused high mortality in the infected pigs, the disease in marine mammals was first reported in 1972 from rectal swabs of Californian sea lions (*Zalophus californianus*) that had just aborted on San Miguel Island (Smith and Boyt, 1990). The causative agent was characterized and named San Miguel sea lion virus type I (SMSV-1). When SMSV-1 was administered in pigs, it caused classical vesicular exanthema syndrome in pigs comparable to the 1932 outbreak (Smith et al., 1973, 1998; Smith and Boyt, 1990). By 1982, calicivirus virus infections had been isolated from 11 pinnipeds and cetaceans and the host range of infected species has continued to increase (Smith et al., 1998). Marine mammal morbilliviruses were first discovered in late 1980s following a large outbreak in which approximately 18,000 harbor seals and gray seals were found dead on Northern European coasts in 1987–1988. The etiological agent was classified as phocine distemper virus (PDV). Similar high mortalities involving thousands of animals were reported in subsequent years in different species including porpoises (*Phocoena phocoena*) (Kennedy et al., 1988), *Phoca caspisa* (Kennedy et al., 2000; Ohashi et al., 2001; Kuiken et al., 2006), *Stenella coeruleoalba* (Domingo et al., 1990), *Tursiops truncates* (Lipscomb et al., 1994a,b), and *Phoca vitulina* (de Swart et al., 1996), harp seals *(Phoca groenlandica*) (Markussen and Have, 1992). The first report of influenza virus infections in marine mammals was in 1979 when more >500 harbor seals were found dead on the North Eastern Coast of the USA due to H7N7 subtype infections (Lang et al., 1981; Webster et al., 1981b). In 1982–1983, an outbreak of influenza due to subtype H4N5 caused mortality in more than 60 harbor seals in Massachusetts coastal areas in the USA (Hinshaw et al., 1984). Since then, influenza A and B viruses have been isolated from different cetaceans and pinnipeds of which postmortem handing has been linked to zoonotic infections in humans (Webster et al., 1981a,b; Hinshaw et al., 1984, 1986).

The earliest reports of adenovirus infections in marine mammals were in the late 1970s in large numbers of sea lions that had clinical signs of hepatitis and enteritis (Britt et al., 1979; Dierauf et al., 1981; Goldstein et al., 2011). Since then, adenoviruses have been isolated from California sea lions, South African fur seals (*Arctocephalus pusillus*), South American sea lions (*Otaria flavescens*) (Inoshima et al., 2013), sei whales (*Balaenoptera borealis*) (Inoshima et al., 2013), bowhead whales (*Balaena mysticetus*) (Smith et al., 1987; Inoshima et al., 2013), beluga whale (*Delphinapterus leucas*), and bottlenose dolphins (*T. truncates*) (Rubio-Guerri et al., 2015). The first report of marine mammal herpesvirus infection was from an outbreak of several harbor seals that had acute pneumonia and hepatitis in the Netherland in 1985 (Osterhaus et al., 1985). Thereafter, herpesviruses have been isolated from several species including harbor seal (*P. vitulina*) (Borst et al., 1986), bottlenose dolphin (*Tursiops truncatus*) (Manire et al., 2006; van Elk et al., 2009), orca (*Orcinus orca*) (Maness et al., 2011), California sea lion (King et al., 2002; Buckles et al., 2006), and gray seals (*Halichoerus grypus*) (Harder et al., 1996). Other viruses shown to infect marine mammals include poxviruses that have been reported in cetaceans and pinnipeds of which some are zoonotic (Van Bressem et al., 1993; Bracht et al., 2006; Waltzek et al., 2012) and astroviruses that have been associated with diarrhea in California sea lions, Steller sea lion (Eumetopias jubatus) and bottlenose dolphin (Rivera et al., 2010). Put together, these studies further consolidate the notion that our response to viral infections in aquatic organisms has mainly been reactive given that the causative agents for these diseases were only identified after they caused massive outbreaks reaching epidemic proportions. Hence, there is need for proactive diagnostic tools for the timely identification of novel pathogens having the potential to cause disease outbreaks in order to help design effective disease control strategies.

## Discovery of new viruses using viral metagenomics

Viral metagenomics has led to discovery of several new viruses in different aquatic organisms including fish, crustaceans, molluscs, turtles, and marine mammals as shown below.

### Novel fish viruses

Recently identified pathogenic viruses of fish include the circovirus isolated from barbell fish (*Barbus barbus*), which causes mortality within 4–6 days after hatching (Lőrincz et al., 2011). Phylogenetic analysis of two complete genomes classified as Barbell circovirus 1 and 2, (BaCV1 and BaCV2) showed that these viruses belong to two new genetic groups within the Circoviridae family, which are distinct from previously known circoviruses (Lőrincz et al., 2011). Apart from barbell fish, circoviruses have been detected in common bream (*Abramis brama*), asp (*Aspius aspius*), round goby (*Neogobius melanostomus*), monkey goby (*Neogobius fluviatilis*), and roach (*Rutilus rutilus*) (Tarján et al., 2014). Novel circoviruses have also been identified from the European catfish (*Silurus glanis*) (Lőrincz et al., 2012) and European eel (*Anguilla anguilla*) showing clinical signs of a cauliflower-like disease (Fichtner et al., 2013). Reuter et al. (2015) identified a novel posavirus designated as Fisavirus 1 (FisaV1) from freshwater carp (*Cyprinus carpio*) and a novel seadornavirus resembling the mammalian Banna virus from freshwater carp (Reuter et al., 2013). In another study, Boros et al. (2011) identified a novel (+)ssRNA virus with a di-cistronic genome in carp while Mor and Phelps (Mor and Phelps, 2016b) identified a novel totivirus from Gold shiner (*Notemigonus crysoleucas*) baitfish using NGS. The majority of viruses shown in Table 3 have only been discovered in the last 4 years unlike viruses in Table 1, which took long to discover using traditional methods. Hence, the rate at which viruses in Table 1 were discovered compared to viruses in Table 3 supports our notion that NGS and viral metagenomics are proactive diagnostic methods that have significantly accelerated our ability to identify novel viruses of fish.

**Table 3:** **Fish and shellfish viruses discovered using NSG and Metagenomics analysis**.

**Virus**	**ABBR**	**Virus family**	**NA**	**Host species (Scientific name)**	Year^*^	**References**
Sea turtle tornovirus 1	STTV1	Papilloma	ssDNA	Florida green sea turtle	2009	Ng et al., 2009a
Erythrocytic necrosis virus	ENV	Iridoviridae	dsDNA	Pacific herring (*Clupea pallasii*)	2011	Emmenegger et al., 2014
Piscine orthoreovirus	PRV	Reoviridae	dsRNA	Atlantic salmon (*Salmo salar* L.)	2011	Palacios et al., 2010
Farfantepenaeus duorarum nodavirus	FdNV	Nodaviridae	(+)ssRNA	Shrimp (*Farfantepenaus duirarum*)	2013	Ng et al., 2013
Catfish circovirus	CfCV	Circoviridae	ssDNA	European Catfish (*Silurus glanuris*)	2013	Lőrincz et al., 2012
Starling circovirus	StCV	Circoviridae	ssDNA	Estuarine Mollusc (*Amphibola crenata*)	2013	Dayaram et al., 2013a
Japanese ell endothelial cell virus	JEECV	Polyomaviridae	dsDNA	Japanese eel (*Anguilla japonica*)	2013	Mizutani et al., 2011
Eel picornavirus	EPV-1	Picornavirus	(+)ssRNA	European eel (*Angilla anguilla*)	2013	Fichtner et al., 2013
Banna virus	BALV	Reoviridae	dsRNA	Fresh water carp (*Cyrpinus carpio*)	2013	Reuter et al., 2013
Gastrop-associated circular ssDNA virus	GaSCV	Unknown	ssDNA	Estuarine mollusk (*Amphibola crenata*)	2013	Dayaram et al., 2013b
Balaton virus	BALV	Reoviridae	dsRNA	Freshwater carp (*Cyrpinus carpio*)	2013	Reuter et al., 2013
Shrimp hepatopancreas associated circular DNA virus	CDV	Circulovirus	ssDNA	Pink shrimp (*Farfantepenaus duirarum*)	2013	Ng et al., 2013
Sea-star wasting disease virus	SSaDV	Parvoviridae	ssDNA	Sea stars (*Pycnopodia helianthoides*)	2014	Hewson et al., 2014
Carp picornavirus 1	CPV-1	Picornaviridae	(+)ssRNA	Carp (*Cyprinus carpio*)	2014	Lange et al., 2014
Densoviruses		Parvoviridae	ssDNA	Urchin (*Colobocentrotus atratus*),	2014	Gudenkauf et al., 2014
Penaeus monodon circovirus 1	PmCV-1	Circoviridae	ssDNA	Shrimp (*Penaeus monodon*)	2014	Pham et al., 2014
Fathead minnow picornavirus	FHMV	Picornaviridae	(+)ssRNA	Golden shiner (*Notemigonus crysoleucas*)	2014	Phelps et al., 2014
Asterias forbesi-associated circular virus	AfaCV	Circulovirus	ssDNA	Asterias forbesi	2015	Fahsbender et al., 2015a
Fisavirus 1	FisaV1	Posavirus	(+)ssRNA	Carp (*Cyrpinus carpio*)	2015	Reuter et al., 2015
White sucker hepatitis B virus	WSHBV	Hepadnaviridae	dsDNA	White sucker (*Catostomus commersonii*)	2015	Hahn et al., 2015
Golden Shiner totivirus	GSTV	Totiviridae	dsRNA	Golden Shiner (*Notemigonus crysoleucas*)	2016	Mor and Phelps, 2016b
Piscine myocarditis like virus	PMCLV	Reoviridae	dsRNA	Golden Shiner (*Notemigonus crysoleucas*)	2016	Mor and Phelps, 2016a
Bluegill hepadnavirus	BGHB	Hepadnaviridae	dsDNA	Bluegill (*Lepomis macrochirus*)	2016	Dill et al., 2016

*NA, Nucleic acid*.

* *Year virus discovered and characterized*.

### Novel viruses of crustaceans

Ng et al. (2013) identified two nodaviruses from the pink shrimp (*Frafantepenaeus duorarum hepatopancreas*), which is a commercially important farmed shrimp species. One virus clone had a 403 nt insert encoding the RNA-dependent-RNA polymerase (RdRp) partial sequence while the other contained a 236 nt insert encoding a capsid protein partial sequence. Phylogenetic analysis showed that the capsid sequence was 43–51% similar to the shrimp nodaviruses, *Macrobrachium rosenbergii* nodavirus (MrNV) and *P. vannamei* nodavirus (PvNV), which causes white tail disease in prawns (*M. rosenbergii*), and muscle necrosis disease in shrimp (*Litopenaeus vannamei*) (Table 2). Apart from FdNV, Ng et al. (2013) also identified the shrimp hepatopancreas associated circular virus (ShrimpCDV) from the digestive tract of the pink shrimp. Based on these findings, it can be concluded that viral metagenomics can be used to identify novel viral pathogens of shrimps. Once the genomic sequence of the novel virus has been determined, specific molecular biology diagnostic tools such as PCR can be designed and used for the screening of brood stock in order to ensure that only SPF stocks are used for shrimp production. In this way viral metagenomics is expected to play an important role in developing effective disease control strategies for novel viral infections of crustaceans before they cause devastating economic losses in aquaculture.

### Novel viruses of mollusks, turtle, and star-fish

Apart from fish and crustaceans, the farming of different species of mollusks, turtle, and starfish has also increased tremendously in the last few decades. Interestingly, novel viruses for these aquatic organisms have also been identified using viral metagenomics (Table 3). Ng et al. (2009a) identified a novel sea turtle tornovirus 1 (STTV1) as the cause of fibropapilloma tumors in Florida sea green turtles. Hewson et al. (2014) identified a novel Forbes sea star (*Asterias forbes*) virus (AFSSV) as the causative agent of a wasting disease condition linked to high mortality in the Forbes sea stars. On the other hand, Andrade et al. (2015) showed that oysters are the hotspots for Mimivirus isolations which are the largest viruses found in the world with >1,100,000 bp length. Overall, these findings show that viral metagenomics analysis is a powerful tool able to identify novel pathogenic viruses infecting different aquatic organisms.

### Novel marine mammal viruses

Captive marine mammals such as the Californian sea lions (*Z. californianus*) and bottle-nose dolphins (*Tursiops tuncatus*) are widely used as recreation animals. And as such, these animals are kept in marine parks where they are used in circus shows and other activities because of their intelligence and easy trainability. Given their close contact with humans, it has become expedient that their disease profile is determined both for public health reasons and for the sake of providing timely healthcare when they become infected with different diseases. Recently, Ng et al. (2009b) used viral metagenomics analysis to identify a novel California sea lion anellovirus (ZcAV) having 35% amino acid homology in the open reading frame (ORF) 1 with the feline anellovirus. In another study, Ng et al. (2011) used metagenomics analysis to identify a novel seal anellovirus (SealAV) in Pacific harbor seals (*Phoca vitulina richardsii*). Kluge et al. (2016) carried out a viral metagenomics survey from feces of subantarctic fur seals (*Arctocephalus tropicalis*) and South America fur seal (*Arctocephalus australis*). From the South America fur seals they identified seal anellovirus 5 (SaV5), and Troque teno salophus virus 1 (TTZV) while in the Subantarctic seal fur picornaviruses were identified namely Fur seal sakobuvirus (FSSV) having 50% amino acid identity resemblance to the *Feline sakobuvirus A* (FSVA). In addition, rotaviruses were identified from the subantarctic fur seals with 45–69% amino acid homology with group C rotaviruses. The identification of rotaviruses from Fur seals has important public health implications given that these animals are commonly found on coastal areas of South America where they attract a lot of tourism where they could serve as a source of zoonotic infections to tourists. These findings suggest that viral metagenomics can be used to obtain disease profiles for aquatic animals as well as profiles of zoonotic pathogens found in marine mammals. Table 4 shows some of the aquatic mammalian viruses discovered using viral metagenomics and NGS. It is interesting to note that most of the viruses shown in Table 3 have only been discovered in the last 4 years further consolidating the notion that viral metagenomics is accelerating our ability to identify novel viruses infecting aquatic organisms.

**Table 4:** **Marine mammalian viruses discovered using NSG and Metagenomics analysis**.

**Virus**	**ABBR**	**Virus family**	**Nucleic acid**	**Host species (Scientific name)**	Year^*^	**References**
California sea lion anellovirus	ZcAV	Anellovirdae	ssDNA	Californian sea lion (*Zalophus californianus*)	2009	Ng et al., 2009b
Steller sea lion reovirus	SSRV	Reoviridae	dsRNA	Steller sea lion (*Eumetopias jubatus*)	2011	Palacios et al., 2011
California sea lion sapovirus	CslSV	Caliciviridae	(+)ssRNA	Californian sea lion (*Zalophus californianus*)	2011	Li et al., 2011
California sea lion sapelovirus	CslSaV	Picornaviridae	(+)ssRNA	Californian sea lion (*Zalophus californianus*)	2011	Li et al., 2011
California sea lion astrovirus	CslAstV	Astroviridae	(+)ssRNA	Californian sea lion (*Zalophus californianus*)	2011	Li et al., 2011
California sea lion norovirus	CslNV1170	Caliciviridae	(+)ssRNA	Californian sea lion (*Zalophus californianus*)	2011	Li et al., 2011
California sea lion rotavirus-1	CslRV1	Reoviridae	dsRNA	Californian sea lion (*Zalophus californianus*)	2011	Li et al., 2011
California sea lion bocaviruses	CslBoV	Parvoviridae	ssDNA	Californian sea lion (*Zalophus californianus*)	2011	Li et al., 2011
Seal parvovirus	SealPV	Parvoviridae	ssDNA	Harbor seals (*Phoca hispida*)	2013	Bodewes et al., 2013
Seal allenovirus 2	SealAV-2	Anelloviridae	(+)ssRNA	Pacific harbor seals (*Phoca vitulina*)	2013	Bodewes et al., 2013
Seal allenovirus 3	SealAV-3	Anelloviridae	(+)ssRNA	Pacific harbor seals (*Phoca vitulina*)	2013	Bodewes et al., 2013
Dolphin rhabdovirus	DRV	Rhabdoviridae	(−)ssRNA	White-beaked dolphin (*Lagenorhynchus albirostris*)	2014	Siegers et al., 2014
Sea lion associated stool parvovirus	Sesavirus	Parvoviridae	ssDNA	Californian sea lion (*Zalophus californianus*)	2015	Phan et al., 2015
Phocine herpesvirus 7	PhHV7	Herpesviridae	dsDNA-RT	Seals (*Phoca vitulina*)	2015	Bodewes et al., 2015
Fur eal sakoburis 1	FSSV)	Pirconaviridae	(+)ssRNA	Subantractic fur seal	2016	Kluge et al., 2016
Seal anellovirus 5	SaV-5	Anelloviridae	(+)ssRNA	Fur seal (*Arctocephalus australis*)	2016	Kluge et al., 2016
Troque teno salophus virus	TTZV 1	Anelloviridae	(+)ssRNA	Fur seal (*Arctocephalus australis*)	2016	Kluge et al., 2016

* *Year virus discovered and characterized*.

## Diagnostic role of viral metagenomics in clinical tissues

Viral metagenomics have been used to directly identify novel etiological agents from tissues of diseased animals showing pathological changes (Finkbeiner et al., 2008; Yongfeng et al., 2011).

### Identification of etiological agents of single infection

Ng et al. (2009a,b, 2013) used lung tissues to identify the ZcAV infecting Sea lions in two separate mortality events. They (Ng et al., 2009b) showed that ZcAV was mainly found in the lungs and pleural-cavity and not in blood, tonsils, lymph nodes, liver, and other organs suggesting that metagenomics can be used to determine tissue tropism of novel viruses in hosts. Follow-up studies showed that the prevalence was high during outbreaks in captive sea lions (100%) and low in reservoir wild sea lions (11%) indicating viral metagenomics can be used to monitor the prevalence of the virus in marine animals. In follow-up studies, the virus from Sea lion was used to develop an enzyme linked immunosorbent assay (ELISA) and specific PCR (Fahsbender et al., 2015b). Apart from ZcAV, Ng et al. (2011) used infected lung tissues of Pacific harbor seals (*P. vitulina* richardsii) to identify SealAV. Hewson et al. (2014) used viral metagenomics to identify the Sea star-associated densovirus (SSaDV) as the causative agent of a wasting disease characterized by behavioral changes, lesions on the limbs, loss of tugor and death due to rapid degradation in infected sea-star (asteroid). They observed that increase in viral titers correlated with increase in pathology in the infected sea stars. Ng et al. (2009a) used tumors to identify STTV1 as the causative agent of fibropappillomas in Florida green sea turtles. Bodewes et al. (2015) identified Phocine herpesvirus 7 (PhHV-7) as the etiological agent of ulcerative gingivitis in phocines (Bodewes et al., 2015) while Enhydralutis papilloma virus 1 (EIPV-1) was shown to be the etiological agent of oral tumors diagnosed in Southern otters (*Enhydra lutris* Nereis) (Ng et al., 2015).

### Identification of etiological agents of co-infections

Yang et al. (2011) have pointed out that viral metagenomics has a high chance of identifying co-infections than traditional diagnostic methods. Ng et al. (2009a) showed that STTV1 existed as a co-infection of a quasispecies of variant strains from a single fibropappilloma tumor in each infected turtle. The STTV1 variants detected by viral metagenomics were identical from the majority part of the viral genome while hypervariable regions (HVRs) were extensively divergent. Based on these findings, it can be concluded that viral metagenomics does not only serve as a diagnostic tool for identifying novel viruses, but it serves as a reliable tool for identifying co-infections of different viruses working together to cause disease as well as identification of co-infections of variant strains of the same virus existing as a quasi-species in a single infection.

## General discussion and conclusions

In this review, we have shown that viral metagenomics is a proactive diagnostic tool able to enhance the discovery of novel pathogenic viruses in aquaculture. In terms of the discovery of novel viruses, the *de novo* assemblies coupled with bioinformatics annotation tools used in viral metagenomics have simplified the task of identifying different sequence segments and variable proteins (VP) that constitute the assemblage of complete viral genomes. As for viral diagnostics, it can be used to identify etiological agents of single infections, co-infections, and tissue tropism. It can also be used for disease surveillance by profiling viruses infecting in different host species and determining disease prevalence in selected host species. As for quasispecies analyses, metagenomics analysis can help identify segments of the viral genome prone to genetic diversity and the conserved segments. Hence, viral metagenomics can be used as multifaceted tool for the identification of novel viruses, phylogenetic analyses, diagnosis of single and co-infection, tissue tropism, and disease surveillance. Moreover, it has been shown that metagenomics analyses can be used to study the epidemiology of viruses outside their susceptible hosts using environmental samples (Munang'andu, 2016). Despite so, viral metagenomics has some limitations that require the support of traditional diagnostic methods. For example, novel viral pathogens identified using viral metagenomics require verification, which calls for isolation of the etiological agent using cell culture, followed by virus characterization and infecting of susceptible hosts to show that the isolated virus is the causative agent of the identified disease by fulfilling the Koch's postulates (Rivers, 1937). And as pointed out by different scientists that the bulk of viruses generated by metagenomics are unculturable (Handelsman, 2004; Schloss and Handelsman, 2005), verification can be a difficult challenge in situation where virus isolation and characterization tools are not available. Although PCR has also been used for verification, Yang et al. (2011) showed lack of correlation of between viral metagenomics and PCR data in their studies, which they attributed to possible errors in annotation, errors *de novo* assembly, sequencing biases and low sequencing depth. These observations further consolidate the notion that verification of viral metagenomics data can be a difficult challenge.

Another important challenge faced in viral metagenomics analysis is that a large proportion of viral sequences generated using this tool remain uncharacterized mainly because they have no similarity with any known sequences in common databanks. Rosario et al. (2009) generated 70% unknown DNA viral sequences from reclaimed water while Zhang et al. (2006) produced 91% unknown viral sequences from human feces. In another study, Breitbart et al. (2002) generated 65% unknown viral sequences from seawater while Rosario et al. (2009) produced 57% unknown RNA viral sequences from reclaimed water. And as pointed out by Mokili et al. (2012), there is a general lack of appropriate bioinformatics tools for the characterization of unknown viral sequences this far. Li et al. (2016), pointed out that one of the problems associated with *de novo* assembly of viral sequences is that they sometimes form chimeric contigs made of artificially combined reads that are not easy to identify. Hence, the bulk of uncharacterized viral sequences, sometimes referred to as the “dark matter of metagenomics,” limits our ability to identify novel pathogens using this tool given that new viruses without homologous sequences in public databases are likely not to be identified. Kim and Bae (2011) compared the linker amplified shotgut library (LASL) with the multiple displacement amplification (MDA) methods and showed that there were more dsDNA viruses amplified from the LASL than the MDA library. On the contrary, the MDA library had more ssDNA viruses than dsDNA viruses from the sample, which is in line with observations made by Roux et al. (2016) who also showed that the choice of amplification method has a bias on ssDNA and dsDNA viruses detected from the same sample. Wommack et al. (2008) showed that short reads (<400 bp) tend to miss distant sequences in phylogenetic classification and that they are prone to miss BLAST homologs found in long reads, indicating that the length of the reads matter thereby posing a challenge in the choice of tools required for *de novo* assembly to ensure that only long reads are produced for easy of taxonomical classification of viruses.

Finally, another important challenge faced with viral metagenomics analysis is that the accuracy of the methods used for aligning the *de novo* assembled genomes have not been investigated in detail (Mokili et al., 2012). The accuracy of annotation methods for identify novel viruses using metagenomics analyses in terms of avoiding false positives and negatives is unknown (Bibby, 2013). Given that the majority of viruses infecting aquatic organisms have not been characterized and that they are not found in databanks, it is highly likely that this inaccuracy might be higher for novel viruses infecting aquatic organisms. Nevertheless, it is evident based on the synopsis put forth in this review that viral metagenomics studies have positively contributed to enhancing our ability to identify novel viruses infecting aquatic organisms. Apart from the discovery of novel pathogens, it is important to point out that viral metagenomics studies have also increased our knowledge of phage dynamics which may new applications for phage therapies (Reyes et al., 2012) suggesting that “viruses are not only demons, but could also serve as angels.”

## Author contributions

All authors listed participated have made substantial, direct and intellectual contribution and approved publication of the manuscript.

### Conflict of interest statement

The authors declare that the research was conducted in the absence of any commercial or financial relationships that could be construed as a potential conflict of interest.
